# CMV-Specific CD8 T Cell Differentiation and Localization: Implications for Adoptive Therapies

**DOI:** 10.3389/fimmu.2016.00352

**Published:** 2016-09-15

**Authors:** Corinne J. Smith, Michael Quinn, Christopher M. Snyder

**Affiliations:** ^1^Department of Microbiology and Immunology, Thomas Jefferson University, Philadelphia, PA, USA

**Keywords:** CMV-specific CD8 T cells, adoptive T cell therapy, tissue-resident memory cells, memory T cells, effector T cells, T cell localization

## Abstract

Human cytomegalovirus (HCMV) is a ubiquitous virus that causes chronic infection and, thus, is one of the most common infectious complications of immune suppression. Adoptive transfer of HCMV-specific T cells has emerged as an effective method to reduce the risk for HCMV infection and/or reactivation by restoring immunity in transplant recipients. However, the CMV-specific CD8^+^ T cell response is comprised of a heterogenous mixture of subsets with distinct functions and localization, and it is not clear if current adoptive immunotherapy protocols can reconstitute the full spectrum of CD8^+^ T cell immunity. The aim of this review is to briefly summarize the role of these T cell subsets in CMV immunity and to describe how current adoptive immunotherapy practices might affect their reconstitution in patients. The bulk of the CMV-specific CD8^+^ T cell population is made up of terminally differentiated effector T cells with immediate effector function and a short life span. Self-renewing memory T cells within the CMV-specific population retain the capacity to expand and differentiate upon challenge and are important for the long-term persistence of the CD8^+^ T cell response. Finally, mucosal organs, which are frequent sites of CMV reactivation, are primarily inhabited by tissue-resident memory T cells, which do not recirculate. Future work on adoptive transfer strategies may need to focus on striking a balance between the formation of these subsets to ensure the development of long lasting and protective immune responses that can access the organs affected by CMV disease.

## Introduction

Severely immunocompromised patients are at great risk from opportunistic infections. These include both new infections acquired from the environment and reactivations of pathogens previously controlled, but not cleared, by the immune system. For over 25 years, investigators have been exploring the potential for adoptive T cell therapy to combat these infections in severely immunocompromised patients. In brief, antiviral T cells are recovered from healthy donors and infused into immunocompromised patients. While this approach is elegant in its simplicity, it is complex in practice and several questions remain: how should the T cells be isolated and selected? Should they be expanded *in vitro* or infused directly? How many T cells are needed? Will the methods used influence the ability of the infused T cells to target the infections at particular sites in the body or persist?

## Cytomegalovirus

One of the most common infectious complications of immune suppression is human cytomegalovirus (HCMV), a ubiquitous β-herpesvirus with a prevalence ranging from 50 to nearly 100% in human populations (1, 2). HCMV causes an acute infection followed by latency that persists for life (3). During the latent stage of infection, HCMV is thought to occasionally reactivate in a stochastic manner and requires continuous control from the host immune system. While HCMV is typically asymptomatic in a healthy host, it is a major clinical concern for immunosuppressed patients (4, 5). In infected HIV patients, HCMV can induce retinitis and less frequently pneumonitis, enterocolitis, or hepatitis (3, 6). HCMV also increases the morbidity and mortality in patients receiving both solid organ transplants and hematopoietic stem cell transplants (HSCT) by increasing the incidence of graft rejection and causing severe organ disease, including pneumonitis, enteritis, hepatitis, pancreatitis, and myocarditis (3, 7–10). Antiviral therapy with gancyclovir and other similar drugs have been successful in decreasing the incidence of HCMV disease, but drug resistance is a growing problem (11). Further, there are several drawbacks to the prolonged use of antiviral drugs, including toxicity to organs, myelosuppression [reviewed in Ref. (12)], and a possible delay in the emergence of HCMV-specific immunity (13, 14).

The need to develop novel anti-HCMV therapies has provided the foundation for developing antiviral adoptive T cell therapies. Numerous studies in HSCT and, more recently, solid organ transplants have shown that adoptive transfer of HCMV-specific T cells from donors reduces the risk for HCMV infection by restoring HCMV immunity, which reduces the need for antiviral therapy and can treat infections that are resistant to antivirals (15–26). Thus, understanding how to optimize the adoptive immunotherapy approach to restore an effective and long-lasting HCMV-specific immunity in patients remains a high priority. The ideal goal of adoptive immune therapy is to transfer T cells that: (1) are capable of immediate and protective effector function, (2) have the ability to localize to the affected organs, and (3) will persist long term. The aim of this review is to briefly summarize the current knowledge about the different CD8^+^ T cell subsets and their functions, particularly in the context of HCMV-specific immunity, and to describe how current adoptive immunotherapy practices might affect the reconstitution of these CD8^+^ T cell subsets in the blood and tissues of patients.

## CMV-Specific T Cell Subsets

### Effector T Cells

Studies have revealed that CMV-specific CD8^+^ T cell populations are heterogeneous mixtures of different subsets with distinct transcriptional profiles, function, and patterns of migration and localization (27–35) (summarized in Table 1). In the case of CMV, the vast majority of CD8s in the blood during latency have a phenotype similar to terminally differentiated effectors (T_EFF_), i.e., high levels of the NK cell inhibitory receptor KLRG1 and low levels of CD127 and CD62L and, in humans, high levels of CD45RA and CD57 (27–32). This phenotype is indicative of repeated antigen stimulation (36, 37); but unlike T cells responding to other chronic infections, CMV-specific T cells do not show signs of functional exhaustion (28, 30–32, 38–41). Indeed, these CMV-specific T cells are cytotoxic and can produce IFN-γ and TNF-α rapidly upon stimulation (28, 30–32, 38).

**Table 1:** **Overview of the major functional differences between CD8 T cell subsets**.

	Phenotype	Immediate effector function	Long-term maintenance	Proliferative capacity	Plasticity	Organ localization
T_EFF_	KLRG1+ CD45RA+	+	−	−	−	−
T_MEM_	CD127+ CD27+ CD45RO+ CD62L+/− CCR7+/−	−	+	+	+	−
T_RM_	CD103+ CD69+	+	+	−	−	+

*Green indicates the presence and red indicates the absence of the indicated attribute*.

Early work in humans as well as the mouse model of murine (M)CMV have noted that CMV-specific T_EFF_ cells accumulated over time after the acute phase of infection was resolved (27, 29–31, 42–44). In fact, an average of 10% of blood CD8s in healthy humans are specific for HCMV epitopes (45). This process was dubbed “memory inflation” (44) and is driven by persistent antigen stimulation (46–49). Interestingly, studies in humans and mice have suggested that CMV-specific inflationary T_EFF_ turn over with a half-life of approximately 45–60 days (32, 38). Moreover, MCMV-specific T_EFF_ do not undergo homeostatic division and have relatively poor proliferative potential (32, 33, 48, 50–52). Thus, the evidence suggests that while CMV-specific T_EFF_ cells are capable of controlling the virus, they are relatively poor at maintaining themselves.

### Memory T Cells

While the majority of CMV-specific T cells in the blood during memory inflation are T_EFF_, a small pool of less differentiated memory T cells (T_MEM_) exists within the inflating CD8 population. These cells express high levels of CD127 and CD27 (and CD45RO in humans) and low levels of KLRG1 and can be further sub-divided into central memory (T_CM_) and effector memory (T_EM_) subsets based on their expression of the lymph node homing molecules CD62L and CCR7 (33, 53). In contrast to terminally differentiated T_EFF_, resting memory T cells are well known to be long-lived, capable of antigen-independent homeostatic division, as well as robust expansion and production of T_EFF_ cells, upon rechallenge (54). This has led to the model that memory inflation is maintained, at least in part, by occasional antigen stimulation of HCMV-specific T_CM_ or T_EM_ subsets, which subsequently divide and differentiate to maintain the large populations of T_EFF_ cells that carry out immune surveillance in latently infected organs (55).

Importantly, we have shown that adoptive transfer of either MCMV-specific T_EFF_ or memory T cells (a mixture of T_CM_ and T_EM_) were sufficient to protect RAG^−/−^ mice from a lethal viral infection. However, MCMV-specific memory T cells could persist long term after transfer had a far greater capacity to expand upon challenge than their T_EFF_ counterparts. Moreover, unlike MCMV-specific T_EFF_ cells, memory T cells could produce both T_EFF_ and new memory T cells after stimulation (33). Thus, while effectors may be sufficient to protect against an immediate viral threat, transplant patients may remain at risk of developing late onset (more than 3 months) or very late onset (more than 1 year) HCMV disease, which is increasingly recognized as a complication of HCMV prophylaxis (56–60). Therefore, long-term protection from disease may rely on reconstituting the full spectrum of HCMV-specific memory T cells.

### Tissue-Resident Memory T Cells

The long held view of T cell migration purports that T_CM_ traffic through the secondary lymphoid organs, while T_EM_ and T_EFF_ cells migrate through the non-lymphoid organs to carry out immune surveillance (53, 61, 62). However, in recent years studies using parabiosis and intravascular staining have revealed that CD8^+^ T cells that remain after a cleared infection only rarely circulate through non-lymphoid organs (63–70). Instead, it has become clear that some T cells in non-lymphoid tissues – particularly mucosal and barrier tissues – adopt yet another differentiation program that enables residency within the tissue, rather than continuous recirculation. Because of their localization, these tissue-resident memory T cells (T_RM_) may play a vital role in the protection of tissues from pathogens. Studies using parabiosis, adoptive transfers, and/or organ transplants have shown that T_RM_ populations are: (1) maintained in the organ long term without recirculating and (2) not replenished by the circulating memory T cell population (61, 68, 69, 71–76). T_RM_ have been identified in many sites throughout a mouse and human, including the skin, liver, lung, brain, sensory ganglia, thymus, kidney, gut, salivary gland, reproductive tract, and even the spleen and blood vessels (77). Tissue-resident T cells have a transcriptional program that is distinct from their circulating counterparts and are frequently identified by expression of CD69 and CD103, although there have been reports of T_RM_ that express neither (70, 78–81).

Because T_RM_ cells are poised at the sites of pathogen entry and reactivation, they provide superior immunity to many local challenges compared to circulating memory cells (71–73, 79, 82–84). Upon antigen stimulation, T_RM_ are typically capable of producing IFN-γ and carrying out cytotoxic activity against infected cells (69, 72, 76, 80, 85–88). In addition, T_RM_-derived IFN-γ induces an antiviral state in the tissue, induces DC maturation and NK cell activation, and recruits circulating T and B cells that can respond to infection (89–91). During MCMV infection, the mucosal organs are primarily occupied by MCMV-specific T_RM_ populations, while the large T_EFF_ populations that characterize memory inflation are mostly restricted to the blood and vasculature (34, 35, 50). However, it is not yet known what role T_RM_ play in the control of MCMV or HCMV latency (34, 35) or in protection from viral disease in immunocompromised patients. The mucosae in general are important sites of persistence, reactivation, and shedding for all herpesviruses; therefore, it is possible that promoting migration of T cells to these sites and promoting their differentiation into T_RM_ could be a major factor in successfully controlling CMV reactivation with adoptive immunotherapy.

### Transcriptional Control of T Cell Differentiation

Each CD8^+^ T cell subset is defined by a transcriptional program that dictates the T cell’s viability, trafficking patterns, and functional capacity (summarized in Table 2). It is well established that the balance between memory and effector CD8 differentiation is controlled by the reciprocal expression of certain transcription factors. In brief, T_EFF_ differentiation is promoted by high expression of T-bet, Blimp-1, and ID2; whereas T_MEM_ formation requires high expression of Eomes, BcL6, and ID3 [reviewed in Ref. (92)]. It is typically thought that promoting one of these subsets antagonizes the development of the other. Less is known about the transcriptional control of T_RM_ differentiation, but recent work is beginning to reveal that it is distinct from both T_EFF_ and T_MEM_ subsets. T_RM_ formation requires downregulation of both Eomes and T-bet (93, 94). However, their long-term survival, at least in the skin and lungs, depends on residual expression of T-bet to drive expression of the IL-15 receptor (94). The establishment of T_RM_ in several organs also depends on the downregulation of KLF2, the transcription factor that is responsible for CD62L expression in T_MEM_. Expression of KLF2 promotes the expression of the S1p1 receptor, which promotes T cell egress from organs and antagonizes CD69 expression and, thus, acts as a switch between circulating and resident memory T cells (95). Additionally, a recently discovered transcription factor, Hobit (Homolog of Blimp1 in T Cells) was specifically upregulated in T_RM_ in mice. T_RM_ formation and maintenance in several organs was dependent on the expression of Hobit in collaboration with Blimp1, while circulating T_MEM_ were unaffected by the loss of both Hobit and Blimp1 (96). However, it is important to note that Hobit expression is not an exclusive marker of T_RM_ cells. In humans, Hobit was highly expressed in CMV-specific effectors isolated from the blood (28, 97). Again, the overlapping and differential use of transcription factors by different T cells subsets suggests that cell fate decisions may be intrinsically linked and mutually exclusive.

**Table 2:** **Transcription factor expression in CD8 T cell subsets**.

	T-bet	Eomes	Bcl6	Blimp-1	Hobit
T_EFF_	++	−	−	++	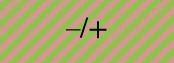
T_MEM_	−	++	++	−	−
T_RM_	+	−	−	+	++

*Red indicates the absence of the indicated transcription factor. Dark green indicates high levels of the indicated transcription factor and light green indicates low levels. The red and green striped panel indicates that transcription factor has been reported to be present in some cases and absent in others*.

## Implications for Adoptive Transfers

### Preserving Memory Capacity

In light of the heterogeneity of the CMV-specific CD8^+^ T cell population, it is important to consider how the source of T cells will impact the efficacy of adoptive immunotherapy. Adoptive immunotherapy typically involves extracting antiviral T cells from the blood. Therefore, in the case of HCMV infection, current transfer protocols will be using mostly T_EFF_ and to a lesser extent circulating T_MEM_ cells. Clinical studies have begun to investigate how the phenotypic make-up of the transferred HCMV-specific CD8 population impacts the outcome of the treatment (18, 98, 99). Peggs et al. showed that rapid CD8^+^ T cell expansion was positively correlated with the number of central memory cells (CD45RA^−^, CCR7^+^) transferred (18). In another study, HCMV-specific CD8s with a memory-like phenotype (CD45RO^+^, CD27^+^, CD57^−^) were more likely to persist and offer protection in a recipient than more differentiated T cells (CD27^−^, CD57^+^) (96). Interestingly, these studies also show that the majority of the CD8s that arise after transfer are terminally differentiated (18, 98). Finally, a non-human primate model of adoptive T cell therapy and CMV infection revealed that donor T cells derived from antiviral T_CM_ cells were more protective that T cells derived from T_EM_ or T_EFF_ sources (100). Thus, it is evident that maintaining the memory potential of adoptively transferred cells may be of critical importance for the eventual success of the therapy.

Maintaining CD8^+^ T cell populations with memory potential during isolation may require unique and carefully controlled conditions. There are a number of factors that promote the formation of T_EFF_ at the expense of T_MEM_, including strong TCR signaling, longer duration of antigen exposure, the presence of pro-inflammatory cytokines during priming (e.g., IL-12, type I IFN, IFN-γ), and repeated antigen stimulation [reviewed in Ref. (101)]. Repeated stimulation is of particular concern due to the fact that most adoptive immunotherapy protocols for selecting antiviral T cells involve antigen stimulation. Short-term peptide stimulation followed by IFN-γ capture has been used to isolate virus-specific T cells (18, 102), and long-term stimulation is commonly used to extensively expand T cells *in vitro* prior to transfer. While T_MEM_ can retain the ability to expand and produce cytokines after multiple challenges, each rechallenge event drives transcriptional changes that leave them less proliferative and more sensitive to terminal differentiation (37, 103, 104). Indeed, in repeated challenge experiments with MCMV, T_MEM_ could recapitulate memory inflation at least through a tertiary challenge; however, the magnitude of inflation and the proportion of CD8s that retained a memory phenotype decreased with each challenge (33). Notably, recent work has suggested that relatively few T cells isolated without expansion using peptide-loaded tetramers (105) or streptamers (99) may be effective at controlling CMV. Eliminating the need for extensive antigen stimulation during adoptive transfer protocols may help to preserve the function and plasticity of HCMV-specific memory cells.

### Migration to the Mucosa

Although many studies have investigated T_CM_, T_EM_, and T_EFF_ differentiation in response to stimulation, it is much less clear how T cells are instructed to traffic to infected tissues and/or develop a T_RM_ (tissue-resident) program. CMV infects and reactivates in several mucosal organs, and HCMV disease after transplant commonly manifests in the lungs and the gut (3). Thus, it is critical to ensure that adoptively transferred antiviral T cells are capable of trafficking to these mucosal sites. Several studies in mice have shown that naive cells, resting memory cells, and highly differentiated effectors have restricted access to non-lymphoid tissues and are not able to respond to T_RM_ differentiation signals *in vivo* or *in vitro* (68, 69, 78, 106–108). Instead, T_RM_ cells form within a short window after infection from recently activated early effectors (68, 69, 74, 78, 106, 107, 109). Consistent with this, adoptive transfer of MCMV-specific CD8s isolated from the spleen at late time points only rarely trafficked to the parenchyma of non-lymphoid organs or differentiated into T_RM_ in latently infected recipients (34). However, when circulating inflationary T cells were transferred into naive mice and challenged, they did form resident memory cells in large numbers in all mucosal organs tested, albeit in lower numbers than MCMV-specific cells derived from naive T cells (34). Additionally, a short *in vitro* peptide pulse was sufficient to increase the migration and differentiation of circulating MCMV-specific T cells into the salivary gland (34). Thus, while at steady state the vast majority of circulating inflationary T cells do not traffic through non-lymphoid tissues or become tissue residents, *in vitro* manipulation prior to adoptive transfer may be able to improve that ability. It is not yet clear what the relative roles of circulating T_EFF_ and T_RM_ are in controlling CMV in non-lymphoid organs either in healthy hosts or in immunocompromised patients. However, in either case, the success of adoptive immunotherapy strategies may depend on ensuring that adoptively transferred T cells can access the afflicted organs and differentiate into T_RM_.

It is also critical to consider how T cells are programmed to migrate into different tissues. T cells can be imprinted with tissue-specific integrins and chemokine receptors during priming, which dictates their subsequent migration pattern. This has been most thoroughly described for the gut, in which retinoic acid expressed by dendritic cells induces the upregulation of the α4β7 integrin and the CCR9 receptor on T cells, which mediates migration to the gut (110). Other examples have been described for the skin and lungs (110–115). Thus, targeting adoptively transferred cells to an organ of interest may require specific manipulation prior to transfer. Alternatively, the recruitment of adoptively transferred T cells to the recipient organ of interest could potentially be improved by treating recipients with chemoattractants. Topical application of the chemokine CXCL9 to the genital tract (108) or the inflammation-inducing hapten DNFB to the skin (82) recruited highly activated circulating effectors T cells to the site. Whether strategies such as these will improve the efficacy of adoptive T cell therapy remains to be studied.

## Summary

The optimal control of HCMV and other opportunistic viral infections depends on establishing a T cell response that incorporates functional effectors, long-term persistence, and the ability to migrate to the sites of viral activity. Cytotoxicity and cytokine production are typically tested for each adoptive transfer protocol reported. However, memory formation and migration to affected organs are also important considerations, and it is not clear how they are affected by typical T cell isolation protocols. Future work needs to identify the factors that will promote the successful and complete reconstitution of HCMV-specific immunity throughout the body and to develop adoptive transfer methods that will optimize these conditions.

## Author Contributions

CJS and MQ wrote the manuscript. CMS and CJS critically discussed and edited the manuscript.

## Conflict of Interest Statement

The authors declare that the research was conducted in the absence of any commercial or financial relationships that could be construed as a potential conflict of interest.
